# Supplementary material for: Patterns of Intron Gain and Loss in Fungi
Source: PLoS Biol. 2004 Nov 30;2(12):e422. doi: 10.1371/journal.pbio.0020422 (PMC532390; doi:10.1371/journal.pbio.0020422)
Supplement: Table S1 — Also available at http://genes.mit.edu/NielsenEtAl/. (4.3 MB ZIP). [file pbio.0020422.st001.zip › NielsenEtAl/html/3.html]

AN4148.1.NCU01132.1.MG04780.1.FG01484.1


```
 CLUSTAL W (1.82) Multiple Sequence Alignments - Introns Inserted


Sequence 1: NCU01132.1	588 aa
Sequence 2: MG04780.1	524 aa
Sequence 3: FG01484.1	550 aa
Sequence 4: AN4148.1	548 aa
Alignment Length: 594 aa
Number Identitical Residues: 230 aa
Alignment Score (without introns) 12057


MG04780.1 	-------MWLTDK-----------------------FYGLRGKSLNYAIVCIA-------
NCU01132.1	MISAYPSTYASDRSVVFLHTSRNKTSFTTSSVAMKPFLGLRGQPLNLAVGAVAGCDFLLF
FG01484.1 	-------MFKK-------------------------YFGLRGQSLNYAISAIAGTDFLLF
AN4148.1  	MTRR--------------------------------YLGLQGQRLQLAISIIAGMDFLLF
          	                                    : **:*: *: *:  :*. .    

MG04780.1 	--D1QGVMGGILTLPVFLSQFPTINPEA---DGLTSIESAQRATNQG~IAVASYNLGCFV
NCU01132.1	GYD1QGVMGGILTLPEFLGYFEQINPDA---PGLTPHESSMRSTYQG~ISVASYNLGCFI
FG01484.1 	GYD1QGVMGGILTMAPFMEQFPDMHSED---PSVSAAVRKNRSNYQG~IAVSSYNLGCFF
AN4148.1  	GYD~QGVTGGLLTLQSFIKYFPTIATNGSYYESLDSAAKSTQSTRQG1IVVAAYNLGCFA
          	. * *** **:**:  *:  *  : .: :   .: .     ::. ** * *::****** 

MG04780.1 	GAVIAIWISNPLGRKRMIILGTSIMVVGTIIKITAFSLVHLVIGRIIMGLG~NGMNTST~
NCU01132.1	GAIITIFIGNPWGRKKIILLGTSIMIVGAILQASATTLGHFIIGRIITGIG~NGGNTST0
FG01484.1 	GAIITIFLGNKLGRKRMIMLGTTIMVIGAALQASAYSLEHFIIGRIITGLG~NGGNTST~
AN4148.1  	GSIPTIWIGNWLGRRKTIFLGSFIMVIGALLQCTSYHLPQFIVGRLVTGFG1NGMNTST~
          	*:: :*::.*  **:: *:**: **::*: :: ::  * ::::**:: *:* ** **** 

MG04780.1 	--VPTWQSETSSSHKRGKMVMIEGALITCGIMISYWIDLGLSFAPG-SVAWRFPIAFQLV
NCU01132.1	SQVPTWQSETSKAHKRGKMVMIEGSLVTAGIMLSYWIDLGLSFAPG-SVAWRFPLAFQII
FG01484.1 	--VPMWQSETCSAHKRGKLVMIEGALITLGITISYWVDYGMYFAQDTSACWRFPMAFQIV
AN4148.1  	--VPTWQSECCKANKRGQLVMIEGAMITCGITISYWIDYGLLFADPNEVAWRFPLAFQIF
          	  ** **** ..::***::*****:::* ** :***:* *: **  ....****:***:.

MG04780.1 	FCFFILAFVWNLPESPRWLILKGHHEEAKLVIAAIADLEVEDNFVQNEFLAIKETVEEMS
NCU01132.1	FCILILIFIPFLPESPRWLVFKGRDAEAKEILAALNDVELDDPIVDTEFHFIHDTVVEMS
FG01484.1 	FCLIIMAFVMNLPESPRWLVLKGRDDDAKEVIAAIADQDAQSKYVDNEFRAMKDTTTEMS
AN4148.1  	FALIILSFVMFLPESPRWLVLKGREDEAREVLGALVGDRSDPSFIQTEFVAIKATVLEMA
          	*.::*: *:  ********::**:. :*: ::.*: .   :   ::.**  :: *. **:

MG04780.1 	KGSFRDLFATNENRNLHRTFLAYLNQVFQQ~ISGINLITYYAAVIYS-GLGMSDFMSRLL
NCU01132.1	KGSFKDLFTMDKDRNFHRTLLAYLNQVFQQ~ISGINLITYYAAVIYS-GLGMSDFLARLL
FG01484.1 	KGAFSDLFSRNDNKNFHRTILAFVNQMFQQ0ISGINLITYYAASIYS-GLGMTGHMPLLM
AN4148.1  	SGSFRDMFTFTEDRHFHRTVLAYVNQMFQQ~ISGINLITYYIPVVLENQLGMNLQNSRLI
          	.*:* *:*:  .::::***.**::**:*** ********** . : .. ***.   . *:

MG04780.1 	AALNGTEYFIASWPAVWLVERVGRRKLMLFGAAGQALTMAASAGVTSRSEEG-FQIAGVV
NCU01132.1	AALNGTEYFIASWPAVFLVERVGRRKLMLFGAIGQAATMAILAGVNSRPDDKPYQIAGIV
FG01484.1 	AALNGTEYFLASLPAIWLVERVGRRKLMLFGAAGQCASMAILAGVGS-SDARACQIVGIV
AN4148.1  	SACNGTEYFLASWIAVFTVEKFGRRTLMLFGAAGMSISMAILAGTAS-AGSNAANIACIV
          	:* ******:**  *:: **:.***.****** * . :**  **. * .   . :*. :*

MG04780.1 	LLFIFNTFFAIGWLGMTWLYPAEIVPLRIRAPANALSTSANWIFNFM0VVMITPVAFNTI
NCU01132.1	FLFVFNTVFAVGWLGMSWLYPAEIVPLRIRAPANALSTSANWIFNFM~VVMITPVAFNKI
FG01484.1 	FLFVFNSFFAVGWLGMTWLYPAEITPLRIRAPANALSTSSNWIFNWL~VVMITPPAFKNI
AN4148.1  	FLFVFNTFFAIGWLGMTWLYPAEIVPLKIRAPANALATSSNWIFNFM~VVMITPVAFDNI
          	:**:**:.**:*****:*******.**:********:**:*****:: ****** **..*

MG04780.1 	KHHTYTIFAII~NAIMVPSVYFLFPETAYRSLEEMDTIFQKVH-GWKGLFTVVRQAEIEP
NCU01132.1	KYQTYIIFAVI~NAFIVPVVYFFYPETACRSLEEMDMIFHKVD-GWKGYFTVVHQAKVEP
FG01484.1 	GSNTYIIFAII~NAIMVPSVYFFFPETAYRSLEEMDTIFQNVDHGLKGALGVVKQAKIEP
AN4148.1  	GYQTYIIFAVI2NAFIFPVTYFFYPETAYRSLEEMDRIFRKTK----SIFSLVRTAKEEP
          	  :** ***:* **::.* .**::**** ******* **::..    . : :*: *: **

MG04780.1 	RRYGKNGELLLDVDAVRAEKGDSS----SAGRENEHREVRTEP-----------------
NCU01132.1	KWYDKDGQRIGGADFEKTAGYQSHSIPESSGFEKPTKAHVESPRADDGITSSSSDGGNRE
FG01484.1 	RRYDNNGNLLIAVEEVEEKAHAEN----RSGRGSSSGQSGEHNN-VAMFTSHDEENQRHE
AN4148.1  	HMYGKHGELMRQLSDVEDEAVRRASYLEHHAGKKEHESSDETSTEKVDNVEHVK------
          	: *.:.*: :   .  .       :  .  .  .                .  .      

MG04780.1 	-
NCU01132.1	S
FG01484.1 	T
AN4148.1  	-
          	
```
